# How Biomedical HIV Prevention Trials Incorporate Behavioral and Social Sciences Research: A Typology of Approaches

**DOI:** 10.1007/s10461-018-2358-0

**Published:** 2018-12-10

**Authors:** Amy Corneli, Karen Meagher, Gail Henderson, Holly Peay, Stuart Rennie

**Affiliations:** 1 Department of Population Health Sciences, Duke University School of Medicine, 215 Morris Street, Suite 210, Durham, NC 27701 USA; 2 Duke Clinical Research Institute, Duke University, Durham, NC USA; 3 Department of Social Medicine, University of North Carolina at Chapel Hill School of Medicine, Chapel Hill, NC USA; 4 RTI International, Research Triangle Park, NC USA

**Keywords:** Biomedical HIV prevention, Behavioral and social sciences research, Clinical trials, Interdisciplinary research

## Abstract

In the field of biomedical HIV prevention, researchers have meaningfully incorporated behavioral and social sciences research (BSSR) into numerous clinical trials, though the timing and degree of integration have been highly variable. The literature offers few frameworks that systematically characterize these collaborations. To fill this gap, we developed a typology of BSSR approaches within biomedical HIV prevention research. Focusing on trials that had safety and/or efficacy endpoints, we identified five approaches for combining BSSR and clinical research: formative, embedded, parallel, explanatory, and implications. We describe each approach and provide illustrative examples. By offering a shared vocabulary for distinguishing the timing and design of collaborative BSSR and clinical research, this typology can facilitate greater transparency in collaborators’ expectations and responsibilities, and help collaborators address challenges likely to be associated with such interdisciplinary research.

**Electronic supplementary material:**

The online version of this article (10.1007/s10461-018-2358-0) contains supplementary material, which is available to authorized users.

## Introduction

Over the past 2 decades, behavioral and social sciences research (BSSR) has increasingly been combined with clinical research in fields as disparate as cancer, heart disease, genetics, and HIV/AIDS [1, 2]. Such collaborations may be peripheral to the clinical research question or essential to understanding contextual factors. Those that are integral to clinical research may focus on assessing the feasibility and acceptability of the intervention, improving the design and conduct of the clinical research, or understanding the environment in which the clinical findings are situated [3].

In the field of HIV prevention, BSSR has been situated both within and alongside clinical research endeavors. It is commonplace for biomedical HIV prevention trials to include substantial BSSR components that contribute to developing the clinical research and understanding the context of the clinical research findings, while also furthering both theoretical and empirical BSSR aims. The impetus for such collaborations is grounded in both science and ethics. The collaborations strengthen the science of clinical research by examining its related sociobehavioral dimensions [4, 5], and they advance ethical norms by incorporating the perspectives and experiences of communities in the design, conduct, and dissemination of clinical research [6, 7].

There has been longstanding US government support for interdisciplinary HIV/AIDS research. Beginning in 1988, the Office of AIDS Research was established to coordinate research across all institutes and centers at the National Institutes of Health (NIH) related to the multifactorial causes and consequences of AIDS [8]. In the same year, interdisciplinary Centers for AIDS Research were funded at US medical institutions [9], many of which include a focus on BSSR research [10]. However, frameworks that systematically characterize interdisciplinary HIV/AIDS research are limited. Recently, Gaist and Stirratt [4] published a framework on HIV/AIDS-related BSSR, highlighting the importance of BSSR research in strengthening the design, conduct, and interpretation of biomedical HIV research [4].

Our article aims to extend Gaist and Stirratt’s framework by providing a typology that illustrates the range of collaborative BSSR and clinical research in biomedical HIV prevention. We first describe the process we used for developing the typology of collaborative approaches. We then provide a description of each approach, elaborating on their different features, such as the objectives and degree of integration in the study protocol. We include examples to illustrate study designs, and when available we describe study findings to demonstrate the richness and utility of data produced from these partnerships. We conclude with suggestions for how the typology can be used, particularly in addressing some of the challenges often associated with interdisciplinary research.

## Developing the Typology

Our development process focused on identifying the breadth of approaches to collaborative BSSR and clinical research by examining a range of case examples. We first created a list of past and current clinical trials in biomedical HIV prevention (i.e., oral pre-exposure prophylaxis [PrEP], vaginal and rectal microbicides, vaginal ring, diaphragm, voluntary medical male circumcision, injectables, monoclonal antibody, and vaccines) by searching for trials on HIV-related network and advocacy websites (e.g., HIV Prevention Trials Network [HPTN], Microbicide Trials Network [MTN], AVAC). For each identified trial, we reviewed the clinical trial protocol for inclusion of any BSSR, if the protocol was publically available (e.g., on the HPTN and MTN websites). We also searched the peer-reviewed literature and HIV/AIDS conference abstracts (i.e., Conference on Retroviruses and Opportunistic Infections [CROI], International AIDS Conference, International AIDS Society Conference on HIV Science) to identify any BSSR linked to a biomedical HIV prevention clinical trial, as well as any BSSR findings related to the clinical research. Lastly, we supplemented this approach by reviewing the peer-reviewed literature of behavioral and social scientists known to work collaboratively with clinical researchers in the biomedical HIV prevention field. We reviewed only BSSR studies related to HIV prevention clinical trials that had safety and/or efficacy endpoints. We excluded (1) trials with only standard quantitative assessments on product acceptability, sexual behavior, and adherence; (2) standalone BSSR related to biomedical HIV prevention or ethics studies in which the primary association with the clinical trial was to gain access to the trial population for research purposes unrelated to the trial’s findings or implications; and (3) open-label studies.

**Fig. 1:**
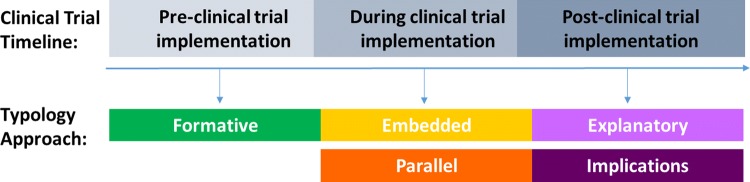
Typology approach by clinical trial tineline

Through reviewing the description of the BSSR components in study protocols, manuscripts, and abstracts, we identified five approaches for conducting BSSR together with biomedical HIV prevention clinical trials, which we categorized as follows: formative, embedded, parallel, explanatory, and implications (Table 1). We also categorized the five approaches by their timing in relation to the conduct of the clinical trial: before, during, and after implementation of the trial (Fig. 1).

**Table 1:** Typology of collaborative behavioral and social sciences research (BSSR) and clinical research in biomedical HIV prevention clinical trials

Approach	Timeline	Objectives	Integration in protocol and consent form(s)	Study population	Funding	Trial examples
Formative	Before clinical trial initiation	1. Determine whether the proposed clinical trial is acceptable to the community and meets their needs 2. Inform clinical and non-clinical-related components of the trial 3. Identify strategies for addressing challenges that arose in prior clinical trials	Separate BSSR protocol and consent form(s)	Prospective trial participants Other key stakeholders	Often same funder as the clinical trial	Breastfeeding, Antiretroviral, and Nutrition (BAN) study Community preparedness for vaccine trials FEM-PrEP (FEM-PrEP Site Preparedness Protocol)
Embedded	During clinical trial implementation	1. Provide context for the clinical trial findings 2. Answer separate but related BSSR questions 3. Inform clinical trial procedures in “real time”	Information about the BSSR and clinical study procedures is *integrated into* a single protocol and consent form(s)	Trial participants	Often same funder as the clinical trial	FEM-PrEP HIVIS03 HPTN 084 IPERGAY MDP 301 MTN 020 MTN 023/ IPM 030 MTN 034/ IPM 045
Parallel	During clinical trial implementation	1. Provide context for the clinical trial findings 2. Answer separate but related BSSR questions 3. Inform future clinical research and rollout	Separate BSSR protocol and consent form(s)	Trial participants Other key stakeholders	Same or different funder from the clinical trial	CAPRISA 004 (The Nested Case–Control Study) Carraguard ÉCLAIR HPTN 035 (HPTN 035A) iPrEx Kenya RCT of male circumcision Partners PrEP VAX 004 VOICE (VOICE C)
Explanatory	After clinical trial implementation	Explain or provide context for clinical trial findings	Separate BSSR protocol and consent form(s), or amendment to an existing protocol	Trial participants Other key stakeholders	Often same funder as the clinical trial	CONRAD Cellulose Sulfate FACTS 001 FEM-PrEP (FEM-PrEP Follow-Up Protocol) Mira VOICE (VOICE D)
Implications	After clinical trial implementation	Explore the behavioral and social implications of the clinical trial findings and/or participation	Separate BSSR protocol and consent form(s)	Trial participants Other key stakeholders	Same or different funder from the clinical trial	HIVIS03

Refer to the manuscript and supplemental tables for details and references

## Typology of BSSR Approaches in Biomedical HIV Prevention Trials

### Before the Trial: Formative

Formative research often uses qualitative data collection methods, such as in-depth interviews and focus group discussions, to gather information from key stakeholders on strategically selected topics to inform the development of clinical research, interventions, or policies. Formative research is typically conducted with potential trial participants and members of their communities before the clinical trial begins. Emphasis is placed on gathering information from individuals who have insights into the preferences of their communities and who may have some influence over trial processes. Formative research is conducted using a separate BSSR protocol and consent form(s) from those used in the clinical trial because the trial is under development. The funding source is often the same. The clinical investigator of the future trial may or may not be included as a collaborator on the formative BSSR protocol.

We identified three objectives of formative research: (1) to determine whether the proposed clinical trial is acceptable to the community and meets the community’s needs; (2) to inform clinical and nonclinical components of the trial, such as study design, recruitment approaches, retention procedures, and adherence counseling; and (3) to identify strategies for addressing challenges that arose in prior clinical trials, such as poor adherence or unexpectedly low HIV incidence in the study population [11]. An example of formative research is provided below, demonstrating objectives 1 and 2. Additional examples are provided in Supplemental Table 1.

Formative research was conducted to inform the study design for the Breastfeeding, Antiretroviral, and Nutrition (BAN) study and to ensure that the proposed clinical trial met the community’s needs. The BAN study was a safety and efficacy clinical trial of a maternal triple-drug antiretroviral regimen or infant nevirapine in reducing postpartum HIV transmission through breastfeeding among women and infants in Malawi [12]. In-depth interviews, focus group discussions, and other data collection approaches were used to gather information from potential trial participants, fathers and grandmothers of small children, and key community informants on infant nutrition, maternal nutrition, and perceptions of study procedures. The formative findings identified several issues that could have jeopardized the the implementation of the trial. Changes were subsequently made to the clinical trial protocol, including extending the time participants received the study drug to account for a longer weaning period, adding a nutritional supplement for infants after breastfeeding cessation, and reducing blood specimen collection [13]. In addition, the formative data revealed many misunderstandings about clinical research among potential trial participants and community members, such as the belief that all study drugs had been previously demonstrated to be safe and efficacious and that randomization was based on individual health needs. These data led to subsequent research to develop and evaluate a context-specific informed consent process for the clinical trial [14, 15].

### During the Trial: Embedded

The embedded approach is one of two approaches in our typology that are implemented concurrently with the clinical trial (Fig. 1). This approach includes the description of both BSSR and clinical study procedures in a single protocol and consent form. The BSSR data collection instruments and planned analyses are also included in a single protocol. Clinical trial data may be used to identify and sample participants for BSSR data collection, and also may be combined with BSSR data to answer the BSSR research questions. Depending on the study design, all or a subset of trial participants are randomly or purposely selected to participate in the BSSR data collection activities, though participants can decline and remain in the clinical trial. Because of this deeply interwoven approach, funding for the BSSR and clinical components are likely provided from the same source, and BSSR scientists are generally listed as collaborators on the study protocol.

We identified 3 objectives of embedded approaches: (1) to understand the broader social and environmental context in which the clinical trial is situated and how that context may affect the clinical trial findings; (2) to answer separate but related BSSR questions; and (3) to inform clinical trial procedures in “real time.” Objectives 1 and 2 are often intertwined. Two examples of embedded research are described below. The FEM-PrEP trial demonstrates objectives 1 and 2, and the Microbicide Development Programme (MPD) 301 study demonstrates objective 3. Additional examples are provided in Supplemental Table 2.

FEM-PrEP was a phase 3 safety and effectiveness trial of tenofovir disoproxil fumarate and emtricitabine (TDF/FTC) for HIV prevention among women in Kenya, South Africa, and Tanzania [16]. Investigators used data on perceptions of HIV risk collected quarterly via quantitative assessments and on drug concentrations in blood measured monthly from trial participants to examine the relationship between HIV risk perceptions and adherence to TDF/FTC in a subsample of the trial population. The researchers reported that having some perception of HIV risk was associated with having good study pill adherence; yet, not all participants with some perception of risk adhered regularly, suggesting that other factors also influenced adherence [17].

A large, comprehensive social science component was embedded in the MDP 301 study, a phase 3, randomized, double-blind, parallel-group clinical trial of 2% PRO2000 and 0.5% PRO2000 among women in Uganda, Tanzania, Zamiba, and South Africa [18]. As part of this component, mixed-methods research was conducted to triangulate data collected on product use and sexual behavior using case report forms, in-depth interviews, and coital diaries [19]. The researchers found that, although participants’ reporting of product use and sexual behavior varied among the three methods of data collection, self-reported data collected through the clinical case report forms were the least reliable data. They also reported that most inconsistences could be reconciled through the triangulation process [20]. A feedback loop was included in the trial protocol for communicating in “real time” the findings from the embedded social science component to the clinical investigators, with an overall aim of potentially making adjustments to data collection or data as needed. Ultimately, attempts to adjust the main clinical trial data set based on the findings of the mixed-methods study were not possible, primarily due to the “rigidity of RCT design” [21].

### During the Trial: Parallel

Similar to embedded BSSR, parallel BSSR is implemented concurrently with the clinical trial. Some objectives are similar, such as (1) to provide context for interpreting the clinical trial’s findings, and (2) to answer separate but related BSSR questions. A third objective can also be present in parallel approaches: to inform implementation of future clinical research and rollout, such as using data to inform future biomedical HIV prevention clinical trials or HIV prevention programs.

Of the two types of BSSR approaches that are conducted concurrently with the clinical trial, parallel approaches typically involve only partial interconnectedness with the clinical research. There are three primary reasons for this. First, the BSSR and clinical research are not combined into a single protocol and consent process. Second, while clinical trial data may be used to identify and sample participants for BSSR data collection, the BSSR data are typically analyzed on their own and not combined with clinical trial data during analyses (see Supplemental Table 3 for an exception). Third, the funding source may be different. Three examples are provided below, demonstrating objective 1 (Partners PrEP), objective 2 (VAX004), and objectives 1 and 3 (Vaginal and Oral Interventions to Control the Epidemic [VOICE]). Additional examples are provided in Supplemental Table 3.

BSSR investigators conducted a qualitative study with Partners PrEP trial participants to better understand motivations for study product adherence among HIV-serodiscordant couples participating in the trial [22]. Partners PrEP was a multisite, phase 3, randomized, double-blind, 3-arm, placebo-controlled trial of daily oral TDF or TDF/FTC for HIV prevention among men and women in serodiscordant relationships in Kenya and Uganda [23]. In-depth interviews were conducted with trial participants from a study site in rural southwestern Uganda and focused on exploring participants’ experiences with adherence to the study drug, as well as the impact serodiscordance had on their relationship. Findings suggested that learning that one partner had HIV but the other did not created a “discordance dilemma” among couples, with the HIV-negative partner expressing concerns about how to stay HIV-negative while remaining in the relationship. Participants perceived PrEP, if proven efficacious for preventing HIV transmission, as a means for couples to stay together. BSSR investigators concluded that relationship dynamics may have influenced adherence decisions, as those who were motivated by a desire to remain in their relationships may be more likely to regularly adhere to the study regimen [22].

In VAX004, a phase 3 clinical trial of an HIV vaccine (AIDSVAX B/B) among women and men in North America and the Netherlands [24], BSSR investigators conducted in-depth interviews with a subset of trial participants (men who have sex with men at 5 US sites) to answer a separate but related social science question—whether participation in an HIV vaccine clinical trial and trial-provided risk reduction counseling influenced participants’ sexual behaviors. Participants viewed counseling positively, though the researchers found that participants’ existing views and practices of risk-taking behavior—grouped as “balancing risks” or “risk homeostasis”—likely determined the influence of risk reduction counseling. For participants in the “balancing risk” group, risk reduction counseling increased their awareness of their risky behaviors and helped to strengthen their already established practice of balancing risk reduction strategies and enjoying their sex life. For participants in the “risk homeostasis” group, risk reduction counseling likely did not influence their sexual behaviors, as these participants had already accepted the potential consequences of their risky sexual behaviors and had previously decided to accept those risks rather than make changes to reduce them [25].

VOICE C was conducted in parallel to the VOICE study, a randomized, placebo-controlled clinical trial to assess daily oral TDF, oral TDF/FTC, and 1% tenofovir vaginal gel for HIV prevention among women in South Africa, Uganda, and Zimbabwe [26]. VOICE C was conducted at the Johannesburg site and aimed to identify contextual factors that influenced adherence. Often such data can also be used to inform future biomedical HIV prevention clinical trials. One VOICE C analysis focused on factors that influenced retention to study clinic visits from a dataset of in-depth interviews, serial interviews, and focus group discussions with trial participants. BSSR investigators found that, among other findings, nondisclosure of trial participation to sexual partners and family members, primarily due to concerns that a negative reaction would result, hindered participants’ ability to keep their study clinic appointments. Family and work obligations were also noted as barriers, as were the length of study visits. The authors stressed the importance of the social context in which trial participants live, and emphasized that future trials should create an active and engaging clinical trial culture that encourages trial retention [27].

### After the Trial: Explanatory

In explanatory approaches, BSSR is conducted after the clinical trial has ended or is coming to an end. The objective is to help explain or provide context for trial findings. Two study designs are common: (1) post-trial BSSR data collection with trial participants that is anticipated within the original clinical trial protocol or added as an amendment to a currently existing protocol; and (2) a separate study with trial participants, and in certain circumstances, with other members of the community. For both explanatory study designs, clinical trial data may be used to identify and sample participants for BSSR data collection (e.g., participants with low or high adherence to the study product). Funding is typically provided by the same source as the clinical trial, and the clinical trial investigator may be included as a collaborator on the explanatory BSSR study. Two examples are provided below, demonstrating study design 1 (FACTS 001) and study design 2 (FEM-PrEP). Additional examples are provided in Supplemental Table 4.

As part of FACTS 001, a phase 3 trial of a pericoital vaginal application of tenofovir 1% gel among women in South Africa, in-depth interviews with trial participants were conducted during their product discontinuation visit in the clinical trial [28]. One purpose of the interviews was to explore understanding about and feasibility of the product regimen. Trial participants reported difficulties following the prescribed regimen, such as the need to conceal gel use from their sexual partners. BSSR investigators concluded that regular use of the gel was not feasible, despite the favorable views of the microbicide gel among participants [29].

In FEM-PrEP [16], a separate, follow-up explanatory study was conducted after closure of the clinical trial to identify reasons trial participants took the study pill, reasons they did not, and reasons for overreporting adherence during the trial. In-depth interviews and audio computer-assisted self-interviews (ACASI) using a quantitative questionnaire were conducted with a subset of trial participants in two sites who were selected based their composite drug adherence scores (a combination of plasma tenofovir and intracellular tenofovir diphosphate drug concentrations) over time. Each participant was shown a picture of her adherence drug concentration scores over time to facilitate discussion of the reasons for adherence/nonadherence. Among participants with moderate and high adherence, five main factors motivated them to take the study pill: (1) participant support of the research, (2) HIV risk reduction (i.e., having hope or an unrealistic belief that the study pill would reduce their HIV risk), (3) pill-taking routines and adherence support tools, (4) adherence counseling, and (5) partner awareness and support [30]. Among participants with none/scarce and moderate adherence, concerns about taking an investigational drug and anxiety about the known or perceived side effects of TDF/FTC, as well as a dislike of the large pill size and daily pill taking, negatively influenced adherence. Participants also described an environment of broader discouragement, where their peers, sexual partners, and community members negatively influenced adherence, primarily due to concerns related to taking an investigational drug, HIV stigma, and perceived side effects [31]. In addition, among all participants, fear that they would be terminated from the trial was the most frequently cited reason for overreporting adherence, reflecting concern about the loss of indirect benefits and ancillary health care they received as trial participants. Other frequently mentioned reasons included not wanting to disappoint trial staff, greater ease of overreporting adherence, and fear of reprimand for nonadherence [32]. VOICE conducted a similar explanatory study, VOICE D, which is described in Supplemental Table 4.

### After the Trial: Implications

Research on broader implications is the second approach in our typology that is conducted after the clinical trial is completed. Using this approach, the BSSR research explores the broader social and behavioral implications of the clinical trial findings and/or of individuals’ participation in the trial. Clinical trial data may be used to identify and sample participants for the BSSR data collection, and a separate BSSR protocol and consent form are likely used. Participants include trial participants but can also include other members of the community. Funding is provided by the same or a different source as the clinical trial, and the clinical trial investigator is likely not included as a collaborator.

As a single example, in a study related to HIVIS03, a phase 1/2 vaccine clinical trial among women and men in Tanzania [33], BSSR investigators administered a structured questionnaire at 2 time points after trial closure to assess the persistence of social harms among a subset of trial participants. Participants reported receiving negative comments from a range of individuals about their trial participation that reflected discrimination, stigma, and mistrust of the vaccine. Workplace colleagues were the primary source of the negative comments, though such comments decreased over time. Participants also reported that some friends, family members, and health care providers also spoke negatively about their involvement in the trial, but these experiences occurred far less often than with workplace colleagues. Investigators recommend that future HIV vaccine trials provide continued support to participants after the trial closes [34].

## Use of the Typology

Our typology of approaches for conducting BSSR research in collaboration with biomedical HIV prevention trials differentiates 2 aspects of partnerships: (1) the timing of the BSSR research (before, during, or after the clinical trial) and (2) the degree of integration of the BSSR and clinical research in study protocols (same or separate protocol/consent form). BSSR conducted before (formative) and after (explanatory and implications) the clinical trial have distinct objectives from BSSR research conducted during the clinical trial (embedded and parallel). While embedded and parallel approaches often have similar objectives, differences in the interconnectedness of BSSR with the clinical trial protocol (same or separate protocol/consent form) may exacerbate or minimize conflict over issues such as data ownership, analysis plans, and leadership. These approaches are also not mutually exclusive. For example, formative BSSR, BSSR conducted during the trial, and post-trial BSSR may be informative and meaningful when applied to a single clinical trial. Explanatory studies can be proposed as components of or extensions to BSSR embedded and parallel studies. Of note, while we present a large number of parallel studies, we observed a trend toward BSSR and clinical research in HIV prevention research endeavors that are increasingly interconnected (e.g., embedded).

This typology has several potential benefits. First, the typology can help in planning future interdisciplinary biomedical research in HIV prevention and other fields, by serving as a tool to generate conversation about key decision points about the primary purpose of the BSSR in the collaboration early, when interdisciplinary teams are designing collaborative BSSR and clinical research. It can help both behavioral and social science researchers and clinical trial investigators anticipate and communicate about the kinds of research questions and data they deem most appropriate to their particular collaboration and for their overall research, especially when some or all may be new to interdisciplinary collaborations. As investigators identify how their own research ideas resemble or differ from the types delineated here, a discussion of the timing, degree of integration, and objectives will provide a constructive way to shape shared expectations. Importantly, these discussions will refine future plans for researchers, including how the BSSR team will practically proceed in conjunction with the clinical trial team. Using the typology will also help to define roles and relationships that govern collection and use of different types of data, and authorship of subsequent publications. It will enable proactive consideration of circumstances that would merit sharing the BSSR findings with the clinical trial team, a practice that may be less common among non-embedded studies and in other fields, and in need of guidance. In summary, the typology can help to identify, anticipate, and minimize some of the practical and ethical challenges that are often associated with interdisciplinary research [3].

Second, the typology may facilitate training and mentoring of the next generation of behavioral and social science researchers and clinical investigators who might be less familiar with the history, process, and contributions of collaborative BSSR and clinical research [35, 36]. Magnus and Castel [37] note a dearth of resources to teach new researchers about team development on a structural level and the need to develop common language as a key communication skill in multidisciplinary HIV research [37].

Third, the typology provides a framework upon which to design and implement evaluations of collaborative BSSR and clinical research. A clearer understanding of study objectives and orientation within the clinical trial process will allow behavioral and social scientists to design more nuanced and meaningful measures of success and impact. Lastly, the typology supports the NIH’s promotion of inter- and multidisciplinary research in HIV/AIDS to address the complexity of pressing public health problems [35, 38, 39].

The proposed typology represents a starting point for ongoing discussion. Future consideration may further nuance our choice of terms for collaborative approaches undertaken during a clinical trial and identify other objectives than those listed here. We selected the terms “embedded” and “parallel” to highlight important differences in interconnectedness—and “explanatory” and “implications” to highlight important differences in objectives—even when the timing of the collaborations is equivalent. We acknowledge that BSSR investigators do not necessarily represent their work with these terms and could have categorized their studies differently from how we did. In addition, we were limited in developing these terms, and assignment of case examples to specific categories, by having access only to information about collaborations provided in the peer-reviewed literature or other sources which are publicly available. Moreover, the examples we provided are not exhaustive of the large body of BSSR conducted in connection with biomedical HIV prevention trials, though we believe they illustrate the range of approaches that have been used to date.

In conclusion, this typology is meant to capture the breadth of interconnectivity of BSSR and clinical research in biomedical HIV prevention research, and offer a finer-grained vocabulary for distinguishing the timing and design of such projects. Documenting the process of integrating BSSR inquiry in biomedical HIV prevention clinical trials and fostering a published discourse about its proceedings can facilitate the growth in quality and diversity of interdisciplinary research. We anticipate fruitful discussion generated by exploring other examples in HIV/AIDS research, leading to new distinctions or visions of collaborations. We also hope that investigators in other areas of interdisciplinary research, such as studies funded by the Ethical, Legal, and Social Implications Program of the National Human Genome Research Institute [40, 41], will suggest areas for improvement, expansion, and refinement of these critical collaborations. As more investigators engage in collaborative BSSR and clinical research and discuss application of this typology, normative differences among the approaches can be identified and further described.

## Electronic supplementary material

Below is the link to the electronic supplementary material.

Supplementary material 1 (DOCX 22 kb)

Supplementary material 2 (DOCX 36 kb)

Supplementary material 3 (DOCX 49 kb)

Supplementary material 4 (DOCX 39 kb)

## References

[1] Lewin S, Glenton C, Oxman AD (2009). Use of qualitative methods alongside randomised controlled trials of complex healthcare interventions: methodological study. BMJ.

[2] O’Cathain A, Thomas KJ, Drabble SJ, Rudolph A, Hewison J (2013). What can qualitative research do for randomised controlled trials? A systematic mapping review. BMJ Open.

[3] Cooper C, O’Cathain A, Hind D, Adamson J, Lawton J, Baird W (2014). Conducting qualitative research within Clinical Trials Units: avoiding potential pitfalls. Contemp Clin Trials.

[4] Gaist P, Stirratt MJ (2017). The roles of behavioral and social science research in the fight against HIV/AIDS: a functional framework. JAIDS.

[5] MacQueen KM (2011). Framing the social in biomedical HIV prevention trials: a 20-year retrospective. J Int AIDS Soc.

[6] UNAIDS. Ethical considerations in biomedical HIV prevention trials. Joint United Nations Programme on HIV/AIDS; 2012.

[7] UNAIDS. Good participatory practice: Guidelines for biomedical HIV prevention trials. Joint United Nations Programme on HIV/AIDS; 2011.

[8] OAR. OAR History: NIH Office of AIDS Research. https://www.oar.nih.gov/about_oar/history.asp. Accessed 1 Oct 2018.

[9] NIAID. Centers for AIDS Research Mission: NIH National Institute of Allergy and Infectious Diseases; updated March 2017. https://www.niaid.nih.gov/research/centers-aids-research-mission. Accessed 1 Oct 2018.

[10] CFAR. Social and Behavioral Science Core: About: University of North Carolina Center for AIDS Research; updated 2017. http://unccfar.org/portfolio/social-behavioral-science/. Accessed 1 Oct 2018.

[11] Padian NS, McCoy SI, Balkus JE, Wasserheit JN (2010). Weighing the gold in the gold standard: challenges in HIV prevention research. AIDS.

[12] Chasela CS, Hudgens MG, Jamieson DJ, Kayira D, Hosseinipour MC, Kourtis AP (2010). Maternal or infant antiretroviral drugs to reduce HIV-1 transmission. N Engl J Med.

[13] Corneli AL, Piwoz EG, Bentley ME, Moses A, Nkhoma JR, Tohill BC (2007). Involving communities in the design of clinical trial protocols: the BAN Study in Lilongwe, Malawi. Contemp Clin Trials..

[14] Corneli AL, Bentley ME, Sorenson JR, Henderson GE, van der Horst C, Moses A (2006). Using formative research to develop a context-specific approach to informed consent for clinical trials. J Empir Res Hum Res Ethics..

[15] Corneli AL, Sorenson JR, Bentley ME, Henderson GE, Bowling JM, Nkhoma J (2012). Improving participant understanding of informed consent in an HIV-prevention clinical trial: a comparison of methods. AIDS Behav.

[16] Van Damme L, Corneli A, Ahmed K, Agot K, Lombaard J, Kapiga S (2012). Preexposure prophylaxis for HIV infection among African women. N Engl J Med.

[17] Corneli A, Wang M, Agot K, Ahmed K, Lombaard J, Van Damme L (2014). Perception of HIV risk and adherence to a daily, investigational pill for HIV prevention in FEM-PrEP. JAIDS..

[18] McCormack S, Ramjee G, Kamali A, Rees H, Crook AM, Gafos M (2010). PRO2000 vaginal gel for prevention of HIV-1 infection (Microbicides Development Programme 301): a phase 3, randomised, double-blind, parallel-group trial. Lancet.

[19] Pool R, Montgomery CM, Morar NS, Mweemba O, Ssali A, Gafos M (2010). A mixed methods and triangulation model for increasing the accuracy of adherence and sexual behaviour data: the microbicides development programme. PLoS ONE.

[20] Pool R, Montgomery CM, Morar NS, Mweemba O, Ssali A, Gafos M (2010). Assessing the accuracy of adherence and sexual behaviour data in the MDP301 vaginal microbicides trial using a mixed methods and triangulation model. PLoS ONE.

[21] Montgomery CM, Pool R (2011). Critically engaging: integrating the social and the biomedical in international microbicides research. J Int AIDS Soc..

[22] Ware NC, Wyatt MA, Haberer JE, Baeten JM, Kintu A, Psaros C (2012). What’s love got to do with it? Explaining adherence to oral antiretroviral pre-exposure prophylaxis for HIV-serodiscordant couples. JAIDS..

[23] Baeten JM, Donnell D, Ndase P, Mugo NR, Campbell JD, Wangisi J (2012). Antiretroviral prophylaxis for HIV prevention in heterosexual men and women. N Engl J Med.

[24] Flynn NM, Forthal DN, Harro DC, Judson FN, Mayer KH, Para MF (2005). Placebo-controlled phase 3 trial of a recombinant glycoprotein 120 vaccine to prevent HIV-1 infection. J Infect Dis.

[25] Guest G, McLellan-Lemal E, Matia DM, Pickard R, Fuchs J, McKirnan D (2005). HIV vaccine efficacy trial participation: men who have sex with men’s experiences of risk reduction counselling and perceptions of risk behaviour change. AIDS Care..

[26] Marrazzo JM, Ramjee G, Richardson BA, Gomez K, Mgodi N, Nair G (2015). Tenofovir-based preexposure prophylaxis for HIV infection among African women. N Engl J Med.

[27] Magazi B, Stadler J, Delany-Moretlwe S, Montgomery E, Mathebula F, Hartmann M (2014). Influences on visit retention in clinical trials: insights from qualitative research during the VOICE trial in Johannesburg, South Africa. BMC Womens Health..

[28] Rees H, Delany-Moretiwe SA, Lombard C, Baron D, Panchia R, Myer L, et al. FACTS 001 phase III trial of pericoital tenofovir 1% gel for HIV prevention in women. Conference on Retroviruses and Opportunistic Infections (CROI); 23–26 February; Seattle, Washington 2015.

[29] Stadler J, Delany-Moretlwe S, Baron D, Ju S, Gray G, Rees H, et al. Adherence to topical PrEP: qualitative findings from the FACTS 001 trial. 21st International AIDS Conference; 18–22, July, 2016; Durban, South Africa 2016.

[30] Corneli A, Perry B, Agot K, Ahmed K, Malamatsho F, Van Damme L (2015). Facilitators of adherence to the study pill in the FEM-PrEP clinical trial. PLoS ONE.

[31] Corneli A, Perry B, McKenna K, Agot K, Ahmed K, Taylor J (2016). Participants’ explanations for nonadherence in the FEM-PrEP clinical trial. JAIDS..

[32] Corneli AL, McKenna K, Perry B, Ahmed K, Agot K, Malamatsho F (2015). The science of being a study participant: FEM-PrEP participants’ explanations for overreporting adherence to the study pills and for the whereabouts of unused pills. JAIDS..

[33] Bakari M, Aboud S, Nilsson C, Francis J, Buma D, Moshiro C (2011). Broad and potent immune responses to a low dose intradermal HIV-1 DNA boosted with HIV-1 recombinant MVA among healthy adults in Tanzania. Vaccine..

[34] Tarimo EA, Munseri P, Aboud S, Bakari M, Mhalu F, Sandstrom E (2014). Experiences of social harm and changes in sexual practices among volunteers who had completed a phase I/II HIV vaccine trial employing HIV-1 DNA priming and HIV-1 MVA boosting in Dar es Salaam, Tanzania. PLoS ONE.

[35] OBSSR. The Office of Behavioral and Social Sciences Research: Strategic Plan 2017-2021. 2016.

[36] Fernandez MI, Wheeler DP, Alfonso SV (2016). Embedding HIV mentoring programs in HIV research networks. AIDS Behav.

[37] Magnus M, Castel A (2016). Breaking down the siloes: developing effective multidisciplinary HIV research teams. AIDS Behav.

[38] Mabry PL, Olster DH, Morgan GD, Abrams DB (2008). Interdisciplinarity and systems science to improve population health: a view from the NIH Office of Behavioral and Social Sciences Research. Am J Prev Med.

[39] Riley WT (2017). Behavioral and social sciences at the National Institutes of Health: methods, measures, and data infrastructures as a scientific priority. Health Psychol.

[40] Henderson GE, Juengst ET, King NM, Kuczynski K, Michie M (2012). What research ethics should learn from genomics and society research: lessons from the ELSI Congress of 2011. J Law Med Ethics..

[41] Sankar PL, Parker LS (2017). The Precision Medicine Initiative’s All of Us Research Program: an agenda for research on its ethical, legal, and social issues. Genet Med..

