# Supplementary material for: How Biomedical HIV Prevention Trials Incorporate Behavioral and Social Sciences Research: A Typology of Approaches
Source: AIDS Behav. 2018 Dec 10;23(8):2146–54. doi: 10.1007/s10461-018-2358-0 (PMC6647486; doi:10.1007/s10461-018-2358-0)
Supplement: Supplementary file 1 — Supplementary material 1 (DOCX 22 kb) [file 10461_2018_2358_MOESM1_ESM.docx]

**Supplemental Table 1. Additional examples of formative approaches**

| **Name of trial** | **Description of the behavioral and social sciences research** |
| --- | --- |
| ***Objective: To determine whether the proposed clinical trial is acceptable to the community and meets the community’s needs*** | |
| Community preparedness for vaccine trials | **Purpose:** To gather data from key stakeholders to inform governmental planning for vaccine trials in Kinshasa, Democratic Republic of Congo.  **Methods:** In-depth interviews and focus group discussions about a hypothetical HIV vaccine trial were conducted with:   - Prospective trial participants. - Community leaders. - University students.   **Findings:**   - Although the findings demonstrated that participants were interested in participating in a vaccine trial—due to an altruistic response to the HIV epidemic in addition to personal protection—numerous significant concerns were raised, including the health risks associated with an experimental vaccine, being tested for HIV, and testing antibody positive because of the vaccine. - The study’s authors raised concerns about proceeding with a vaccine trial until more education on HIV and clinical trials could be provided within the community and until trust between researchers and the community could be established. - A vaccine trial was not initiated [1]. |
| ***Objective: To identify strategies for addressing challenges that arose in prior clinical trials*** | |
| FEM-PrEP, a phase III safety and effectiveness trial of tenofovir disoproxil fumarate and emtricitabine (TDF/FTC) for HIV prevention among women in sub-Saharan Africa [2] | **Background:** HIV incidence was discovered to be too low among women in sub-Saharan Africa participating in a clinical trial conducted by FHI 360 to test the efficacy of tenofovir disoproxil fumarate as PrEP [3]. To ensure that populations at high risk of HIV infection were enrolled in FHI 360’s subsequent PrEP trial—FEM-PrEP—sites known to have high HIV incidence were selected, and formative research was conducted to focus recruitment efforts.  **Purpose:** To identify places where new sexual partnerships are formed (FEM-PrEP Site Preparedness Protocol).  **Methods:** The PLACE method [4].  **Findings:**   - Public places were people met sex partners were identified. - Results were used to prioritize recruitment efforts in areas where the incidence of HIV might be high in two FEM-PrEP sites, Bondo and Pretoria [5]. |

**References:**

1. Olin J, Kokolamami J, Lepira FB, Mwandagalirwa K, Mupenda B, Ndongala ML, et al. Community preparedness for HIV vaccine trials in the Democratic Republic of Congo. Cult Health Sex. 2006;8(6):529-44.
2. Van Damme L, Corneli A, Ahmed K, Agot K, Lombaard J, Kapiga S, et al. Preexposure prophylaxis for HIV infection among African women. N Engl J Med. 2012;367(5):411-22.
3. Peterson L, Taylor D, Roddy R, Belai G, Phillips P, Nanda K, et al. Tenofovir disoproxil fumarate for prevention of HIV infection in women: a phase 2, double-blind, randomized, placebo-controlled trial. PLoS Clin Trials. 2007;2(5):e27.
4. Weir SS, Pailman C, Mahlalela X, Coetzee N, Meidany F, Boerma JT. From people to places: focusing AIDS prevention efforts where it matters most. AIDS. 2003;17(6):895-903.
5. Parker C, Corneli A, Agot K, Odhiambo J, Asewe J, Ahmed K, et al. Lessons learnt from implementing an empirically informed recruitment approach for FEM-PrEP, a large HIV prevention clinical tiral. Open Access J Clin Trials. 2015;7:1-9.
