# Supplementary material for: How Biomedical HIV Prevention Trials Incorporate Behavioral and Social Sciences Research: A Typology of Approaches
Source: AIDS Behav. 2018 Dec 10;23(8):2146–54. doi: 10.1007/s10461-018-2358-0 (PMC6647486; doi:10.1007/s10461-018-2358-0)
Supplement: Supplementary file 2 — Supplementary material 2 (DOCX 36 kb) [file 10461_2018_2358_MOESM2_ESM.docx]

**Supplemental Table 2. Additional examples of embedded approaches**

| **Name of trial** | **Description of the behavioral and social sciences research^1,2^** |
| --- | --- |
| ***Objective: To provide context for the clinical trial findings and/or to answer separate but related behavioral and social sciences research questions*** | |
| HIVIS03—a phase I/II vaccine clinical trial [1] | **Purpose:** To explore reasons trial participants did not continue with their planned investigational vaccinations.  **Methods:** Qualitative interviews were conducted with participants who withdrew after randomization.  **Findings:** Participants:   - Described fears of receiving an investigational vaccine, including the potential for unknown side effects or concerns it may harm their reproductive health. - Described resistance received from others. - Expressed concern that taking the investigational vaccine could harm their current social bonds, among other worries. - Suggested that trial investigators provide education about HIV vaccine to the general public and involve participants’ significant others, as appropriate, from the start of the trial [2]. |
| Microbicide Trial Network [MTN] 020—a phase III clinical trial to evaluate the safety and efficacy of dapivirine ring among women in sub-Saharan Africa [3,4]^3^ | **Purpose:** To assess the acceptability of the vaginal ring, among a variety of topics.  **Methods:**   - Single in-depth interviews (IDIs) were conducted with early product discontinuers (mostly due to seroconversion). - Up to three serial IDIs were conducted with a purposefully-selected sub-set of trial participants (based on adherence experiences). - Focus group discussions (FGDs) were conducted with trial participants.   **Findings:** In one analysis, three main findings related to vaginal ring acceptability were reported:   - Participants’ initial concerns about the vaginal ring diminished over time. - Support from staff and peers helped participants to overcome challenges using the vaginal ring. - Male partners likely had the most influence on participants’ use and acceptability of the vaginal ring [5]. |
| MTN-023/International Partnership for Microbicides (IPM) 030—A phase 2a safety study of the Dapivirine vaginal ring in adolescent females [6] | **Purpose:** To understand participants’ experiences in using the vaginal ring, factors that influenced ring adherence, such as participants’ home environments and partner perspectives, and vaginal hygiene practices.  **Methods:** IDIs with a sub-set of trial participants. |
| ANRS IPERGAY—A clinical trial on the efficacy of “on demand” antiretroviral PrEP (TDF/FTC) for HIV prevention among men who have sex with men [7-9] | **Purpose:** To understand trial participants’ motivations, difficulties, sexual behaviors, expectations, and adherence.  **Methods:** Quantitative questionnaires, and serial IDIs with trial participants, starting at enrollment and ending at the conclusion of the trial; FGDs [10]. |
| MTN-034/IPM 045—A phase 2a crossover trial evaluating the safety of and adherence to the Dapivirine vaginal ring and TDF/FTC in adolescents and young adult females [11] | **Purpose:** To gain additional insight into barriers to product adherence, motivation for joining the clinical trial, and sexual practices before and when using the study product.  **Methods:** IDIs and FGDs with trial participants. |
| HPTN 084—A phase 3 double blind safety and efficacy study of long-acting injectable Cabotegravir compared to daily oral TDF/FTC among women [12] | **Purpose:** To explore 1) participants’ perceptions of trial participation, and acceptability of and experiences with using the study product, and 2) sexual partners’ experiences related to the trial.  **Methods:**   - Single or serial IDIs and/or FGDs with trial participants. - IDIs and FGDs with male sexual partners (tentative). |
| ***Objective: To inform clinical trial procedures in “real-time”*** | |
| MDP 301—A phase III, randomized, double-blind, parallel-group clinical trial of 2% PRO2000 and 0.5% PRO2000 [13] | **Purpose:** To understand how trial participants understood the justification for closing the 2% PRO2000 gel arm of the MDP 301 clinical trial early (because of futility) but continuing within the 0.5% PRO2000 gel arm.  **Method:**   - IDIs and FGDs with trial participants discontinued from the 2% PRO2000 study arm. - Field notes of conversations observed among participants at the study clinic.   **Findings:**   - Concerns stemming from the interview notification process and misperceptions of the trial-provided explanation were quickly identified and led to the immediate modification of the script that trial staff subsequently used to explain the rationale for the early closure. - Social scientists reported that integrating findings from social and behavioral science into trial procedures in real-time eliminated participants’ concerns and misperceptions during the remaining dissemination activities [14]. |

^1^Findings are reported if available.

^2^The BSSR studies may have had other objectives than those listed here.

^3^The qualitative component was not initially embedded but was added as an amendment to the clinical trial protocol.

**References:**

1. Bakari M, Aboud S, Nilsson C, Francis J, Buma D, Moshiro C, et al. Broad and potent immune responses to a low dose intradermal HIV-1 DNA boosted with HIV-1 recombinant MVA among healthy adults in Tanzania. Vaccine. 2011;29(46):8417-28.
2. Tarimo EA, Thorson A, Kohi TW, Bakari M, Mhalu F, Kulane A. Reasons for declining to enroll in a phase I and II HIV vaccine trial after randomization among eligible volunteers in Dar es Salaam, Tanzania. PLoS One. 2011;6(2):e14619.
3. Baeten JM, Palanee-Phillips T, Brown ER, Schwartz K, Soto-Torres LE, Govender V, et al. Use of a vaginal ring containing dapivirine for HIV-1 prevention in women. N Engl J Med. 2016;375(22):2121-32.
4. MTN-020. MTN-020 Qualitative: Microbicide Trials Network. <http://www.mtnstopshiv.org/node/4651>. Accessed 1 Oct 2018.
5. Montgomery ET, van der Straten A, Chitukuta M, Reddy K, Woeber K, Atujuna M, et al. Acceptability and use of a dapivirine vaginal ring in a phase III trial. AIDS. 2017;31(8):1159-67.
6. MTN-023. MTN-023/IPM 030 Phase 2a Safety Study of a Vaginal Ring Containing Dapivirine in Adolescent Females: Microbicide Trials Network; 2015. <http://www.mtnstopshiv.org/sites/default/files/attachments/MTN-023%20IPM%20030%20Version%202%200_14Jan2015_Updated.pdf>. Accessed 1 Oct 2018.
7. Molina JM, Charreau I, Spire B, Cotte L, Chas J, Capitant C, et al. Efficacy, safety, and effect on sexual behaviour of on-demand pre-exposure prophylaxis for HIV in men who have sex with men: an observational cohort study. Lancet HIV. 2017;4(9):e402-e10.
8. Molina JM, Capitant C, Spire B, Pialoux G, Cotte L, Charreau I, et al. On-demand preexposure prophylaxis in men at high risk for HIV-1 infection. N Engl J Med. 2015;373(23):2237-46.
9. Molina J-M, Capitant C, Spire B, Pialoux G, Cotte L, Charreau I, et al. Protocol for: Molina J-M, Capitant C, Spire B, et al. On-demand preexposure prophylaxis in men at high risk for HIV-1 infection. N Engl J Med 2015; 373:2237-46. Prepwatch.org. <https://www.prepwatch.org/wp-content/uploads/2016/11/IPERGAY_protocol.pdf>. Accessed 1 Oct 2018.
10. ANRS IPERGAY Study protocol. <https://www.prepwatch.org/wp-content/uploads/2016/11/IPERGAY_protocol.pdf>. Accessed 1 Oct 2018.
11. MTN-034. MTN-034/IPM 045 A Phase 2a Crossover Trial Evaluating the Safety of and Adherence to a Vaginal Matrix Ring Containing Dapivirine and Oral Emtricitabine/Tenofovir Disoproxil Fumarate in an Adolsecent and Young Adult Female Population: Microbicide Trials Network; 2017. <http://www.mtnstopshiv.org/sites/default/files/attachments/MTN-034_IPM%20045_Version%201.0_2Feb2017_FINAL_0.pdf>. Accessed 1 Oct 2018.
12. HPTN-084. HPTN 084: A Phase 3 Double Blind Saftey and Efficacy Study of Long-Acting Injectable Cabotegravir Compared to Daily Oral TDF/FTC for Pre-Exposure Prophylaxis in HIV-Unifected Women: HIV Prevention Trials Network; 2017. <https://www.hptn.org/sites/default/files/inline-files/HPTN%20084%20Protocol%20FINAL%20Version%201.0%2C%202March2017_0.pdf>. Accessed 1 Oct 2018.
13. McCormack S, Ramjee G, Kamali A, Rees H, Crook AM, Gafos M, et al. PRO2000 vaginal gel for prevention of HIV-1 infection (Microbicides Development Programme 301): a phase 3, randomised, double-blind, parallel-group trial. Lancet. 2010;376(9749):1329-37.
14. Gafos M, Mzimela M, Ndlovu H, Mhlongo N, Hoogland Y, Mutemwa R. "One teabag is better than four": participants response to the discontinuation of 2% PRO2000/5 microbicide gel in KwaZulu-Natal, South Africa. PLoS One. 2011;6(1):e14577.
