# Supplementary material for: How Biomedical HIV Prevention Trials Incorporate Behavioral and Social Sciences Research: A Typology of Approaches
Source: AIDS Behav. 2018 Dec 10;23(8):2146–54. doi: 10.1007/s10461-018-2358-0 (PMC6647486; doi:10.1007/s10461-018-2358-0)
Supplement: Supplementary file 3 — Supplementary material 3 (DOCX 49 kb) [file 10461_2018_2358_MOESM3_ESM.docx]

**Supplemental Table 3. Additional examples of parallel approaches**

| **Name of trial** | **Description of the behavioral and social sciences research^1^** |
| --- | --- |
| ***Objective: To provide context for the clinical trial findings*** | |
| VOICE—a randomized, placebo-controlled clinical trial to assess daily oral TDF, oral TDF/FTC, and 1% tenofovir vaginal gel for HIV prevention among women in South Africa, Uganda, and Zimbabwe [1] | **Purpose:** To explore socio-cultural and contextual factors that influenced participants’ use of the study products among participants at the Johannesburg site (VOICE C).  **Methods:** The following were conducted with trial participants:   - In-depth interviews (IDIs). - Serial ethnographic interviews. - Focus group discussions (FGDs).   **Findings:** Three major themes were identified:   - Ambivalence toward research. - Preserving a healthy status. - Managing social relationships [2].   **Purpose:** To explore male influence on product use at the Johannesburg site (VOICE C).  **Methods:** IDIs, serial ethnographic interviews, and FGDs were conducted with trial participants, and IDIs and FGDs were conducted with male partners of trial participants.  **Findings:** Male partners’ level of understanding or misunderstanding about the purpose of the VOICE trial, as well as their overall support of their partner as a trial participant and acknowledgement of shifting power roles, may have influenced participants’ use of the study products [3]. |
| iPrEx—A clinical trial to evaluate the safety and efficacy of once-daily oral TDF/FTC among men and transgender women who have sex with men [4] | **Purpose:** To provide insights into participants’ experiences as trial participants, as well as the individual and contextual factors influencing their use of the study product.  **Methods:** IDIs and FGDs were conducted with a sub-sample of participants from the Chiang Mai, Thailand site, during the implementation of the iPrEx trial  **Findings:** Participants reported that the following influenced adherence to the study pill:   - Individual-level factors, such as positive beliefs about the study product and medication management skills. - Interpersonal-level factors, such as support from family and friends [5].   **Methods:** IDIs and FGDs were conducted with a sub-sample of participants from the San Francisco site, before and after announcing the study results  **Findings:** Among other findings, participants:   - Reported that a sense of altruism motivated their adherence to the study product. - Described the importance of patient-centered counseling and care. - Identified several “daily life” barriers to adherence, such as changes in their routine or a busy schedule [6]. |

| **Name of trial** | **Description of the behavioral and social sciences research^1^** |
| --- | --- |
| ***Objective:*** ***To answer separate but related behavioral and social sciences research questions*** | |
| The Kenya randomized clinical trial on voluntary medical male circumcision [7] | **Purpose:** To assess risk compensation (previous analyses suggested that trial participants did not increase their risky sexual behaviors after male circumcision; however, investigators of the parallel study noted that those analyses were conducted using limited sexual behavioral measures that were collected as part of the clinical trial dataset).  **Methods:** A comprehensive set of sexual behavioral measures were collected.  **Findings:** The analysis did not demonstrate that participants increased their sexual risk behavior after circumcision; in fact, participants significantly reduced their risky sexual behaviors for the 12 months after the procedure [8]. |
| HPTN 035—a phase II/IIb double-blind randomized controlled trial of BufferGel and 0.5% PRO 2000 Gel [9] | **Purpose:** To explore comprehension of the trial’s informed consent process and motivations for joining the trial (HPTN 035A).  **Methods:** IDIs were conducted with trial participants and male partners.  **Findings:**   - Social scientists identified narratives suggesting that participants may have had preventive misconceptions [10]:^2^ - Participants described joining the trial to “protect myself” or “prevent HIV infection,” in addition to reasons such as altruism and having access to HIV testing and counseling. - Some participants stated that they would be protected from HIV if the gel was shown to be efficacious, although they did not acknowledge that they may have been assigned the placebo. - Findings led the authors to coin a new term, “logical preventive misconception” [11]. |
| Partners PrEP—A multi-site, phase III, randomized, double-blind, three-arm, placebo-controlled trial of daily oral TDF or TDF/FTC for HIV prevention among men and women in serodiscordant relationships [12] | **Purpose:** To better understand factors that led to inconsistent condom use during the trial, as well as how couples were coping with becoming a seroconcordant couple.  **Methods:** Individual IDIs were conducted with participants who seroconverted during the clinical trial and with their sexual partners at one of the Kenyan sites.  **Findings:**   - Alcohol use, low risk perceptions due to multiple HIV negative tests, and limited acceptability of condoms contributed to inconsistent condom use. - Among those learning their HIV-positive status, participants who seroconverted reported both negative (e.g., increased alcohol use) and positive (e.g., increased partner support) coping strategies. - Authors conclude that couples counseling will be important to promote positive coping strategies and adherence to ART and/or PrEP [13]. |

| **Name of trial** | **Description of the behavioral and social sciences research^1^** |  |
| --- | --- | --- |
| CAPRISA 004—a two-arm, double-blind, randomized, placebo-controlled clinical trial of 1% tenofovir gel among women in South Africa (14)^3^ | **Purpose:** To model potential HIV exposure with adherence to the study gel and examine patterns of gel use (The Nested Case-Control Study).  **Methods:**   - Trial participants who seroconverted were invited to enroll in the nested case/control study as cases within three weeks of testing HIV positive. - Participants who tested negative were randomly selected continuously during trial implementation and invited to participate as controls. - BSSR investigators collected comprehensive three-month recall data on sexual events and gel use among cases and controls using the time-line follow-back method, documenting single versus double dosing (per protocol) of the gel for each sex act, and analyzed those data together with clinical trial data on HIV seroconversion.   **Findings:**   - No meaningful association between self-reported gel use and risk of HIV was found. - Operationally, the authors concluded that it is feasible to conduct a rigorous social science research study within clinical research (15). |  |
| ***Objective: To inform future clinical research and rollout*** | | |
| The phase II expanded safety trial of the Carraguard microbicide gel [16] | **Purpose:** To explore trial participants’ experiences as trial participants to inform the phase III trial.  **Method:** FGDs  **Findings:**   - Participants described beneficial aspects of trial participation, such as their increased understanding of HIV transmission. - Participants indicted areas for improvement in trial conduct, such as providing contraception [17]. - Findings were used to inform the implementation of the phase III trial of the Carraguard microbicide gel [18]. | |
| ECLAIR—A double-blind, randomized, multi-center phase 2 safety and acceptability trial of the injectable CAB-LA [19] | **Purpose:** To explore participants’ experiences with an investigational injectable for HIV prevention, to inform the phase III trial.  **Methods:** IDIs were conducted with a sub-sample of trial participants.  **Findings:**   - Five themes were identified, primarily describing participants’ experiences of pain and anxiety. - Social scientists described how these data can inform 1) components of the subsequent phase 3 clinical trial of CAB-LA, and 2) patient education and provider guidance on injectable PrEP, if demonstrated to be safe and efficacious [20]. | |

^1^The BSSR studies may have had other objectives than those listed here.

^2^Preventive misconception is the belief that participants will benefit personally from trial participation—i.e., help them to remain HIV negative.

^3^An example of using clinical and BSSR data in a combined analysis.

**References:**

1. Marrazzo JM, Ramjee G, Richardson BA, Gomez K, Mgodi N, Nair G, et al. Tenofovir-based preexposure prophylaxis for HIV infection among African women. N Engl J Med. 2015;372(6):509-18.
2. van der Straten A, Stadler J, Montgomery E, Hartmann M, Magazi B, Mathebula F, et al. Women's experiences with oral and vaginal pre-exposure prophylaxis: the VOICE-C qualitative study in Johannesburg, South Africa. PLoS One. 2014;9(2):e89118.
3. Montgomery ET, van der Straten A, Stadler J, Hartmann M, Magazi B, Mathebula F, et al. Male partner influence on women's HIV prevention trial participation and use of pre-exposure prophylaxis: the importance of “understanding". AIDS Behav. 2015;19(5):784-93.
4. Grant RM, Lama JR, Anderson PL, McMahan V, Liu AY, Vargas L, et al. Preexposure chemoprophylaxis for HIV prevention in men who have sex with men. N Engl J Med. 2010;363(27):2587-99.
5. Tangmunkongvorakul A, Chariyalertsak S, Amico KR, Saokhieo P, Wannalak V, Sangangamsakun T, et al. Facilitators and barriers to medication adherence in an HIV prevention study among men who have sex with men in the iPrEx study in Chiang Mai, Thailand. AIDS Care. 2013;25(8):961-7.
6. Gilmore HJ, Liu A, Koester KA, Amico KR, McMahan V, Goicochea P, et al. Participant experiences and facilitators and barriers to pill use among men who have sex with men in the iPrEx pre-exposure prophylaxis trial in San Francisco. AIDS Patient Care STDS. 2013;27(10):560-6.
7. Bailey RC, Moses S, Parker CB, Agot K, Maclean I, Krieger JN, et al. Male circumcision for HIV prevention in young men in Kisumu, Kenya: a randomised controlled trial. Lancet. 2007;369(9562):643-56.
8. Mattson CL, Campbell RT, Bailey RC, Agot K, Ndinya-Achola JO, Moses S. Risk compensation is not associated with male circumcision in Kisumu, Kenya: a multi-faceted assessment of men enrolled in a randomized controlled trial. PLoS One. 2008;3(6):e2443.
9. Abdool Karim SS, Richardson BA, Ramjee G, Hoffman IF, Chirenje ZM, Taha T, et al. Safety and effectiveness of BufferGel and 0.5% PRO2000 gel for the prevention of HIV infection in women. AIDS. 2011;25(7):957-66.
10. Simon AE, Wu AW, Lavori PW, Sugarman J. Preventive misconception: its nature, presence, and ethical implications for research. Am J Prev Med. 2007;32(5):370-4.
11. Woodsong C, Alleman P, Musara P, Chandipwisa A, Chirenje M, Martinson F, et al. Preventive misconception as a motivation for participation and adherence in microbicide trials: evidence from female participants and male partners in Malawi and Zimbabwe. AIDS Behav. 2012;16(3):785-90.
12. Baeten JM, Donnell D, Ndase P, Mugo NR, Campbell JD, Wangisi J, et al. Antiretroviral prophylaxis for HIV prevention in heterosexual men and women. N Engl J Med. 2012;367(5):399-410.
13. Ngure K, Vusha S, Mugo N, Emmanuel-Fabula M, Ngutu M, Celum C, et al. "I never thought that it would happen ... " Experiences of HIV seroconverters among HIV-discordant partnerships in a prospective HIV prevention study in Kenya. AIDS Care. 2016;28(12):1586-9.
14. Abdool Karim Q, Abdool Karim SS, Frohlich JA, Grobler AC, Baxter C, Mansoor LE, et al. Effectiveness and safety of tenofovir gel, an antiretroviral microbicide, for the prevention of HIV infection in women. Science. 2010;329(5996):1168-74.
15. MacQueen KM, Weaver MA, van Loggerenberg F, Succop S, Majola N, Taylor D, et al. Assessing adherence in the CAPRISA 004 tenofovir gel HIV prevention trial: results of a nested case-control study. AIDS Behav. 2014;18(5):826-32.
16. Carraguard Phase II South Africa Study Team. Expanded safety and acceptability of the candidate vaginal microbicide Carraguard(R) in South Africa. Contraception. 2010;82(6):563-71.
17. Pistorius AG, van de Wijgert JH, Sebola M, Friedland B, Nagel E, Bokaba C, et al. Microbicide trials for preventing HIV/AIDS in South Africa: phase II trial partricipants' experiences and psychological needs. SAHARA J. 2004;1(2):78-86.
18. Skoler-Karpoff S, Ramjee G, Ahmed K, Altini L, Plagianos MG, Friedland B, et al. Efficacy of Carraguard for prevention of HIV infection in women in South Africa: a randomised, double-blind, placebo-controlled trial. Lancet. 2008;372(9654):1977-87.
19. Markowitz M, Frank I, Grant RM, Mayer KH, Elion R, Goldstein D, et al. Safety and tolerability of long-acting cabotegravir injections in HIV-uninfected men (ECLAIR): a multicentre, double-blind, randomised, placebo-controlled, phase 2a trial. Lancet HIV. 2017;4(8):e331-e40.
20. Meyers K, Rodriguez K, Brill AL, Wu Y, La Mar M, Dunbar D, et al. Lessons for patient education around long-acting injectable PrEP: findings from a mixed-method study of phase II trial participants. AIDS Behav. 2018; 22(4):1209-16.
