# Supplementary material for: How Biomedical HIV Prevention Trials Incorporate Behavioral and Social Sciences Research: A Typology of Approaches
Source: AIDS Behav. 2018 Dec 10;23(8):2146–54. doi: 10.1007/s10461-018-2358-0 (PMC6647486; doi:10.1007/s10461-018-2358-0)
Supplement: Supplementary file 4 — Supplementary material 4 (DOCX 39 kb) [file 10461_2018_2358_MOESM4_ESM.docx]

**Supplemental Table 4. Additional examples of explanatory approaches**

| **Name of trial** | **Description of the behavioral and social sciences research^1^** |
| --- | --- |
| VOICE—a randomized, placebo-controlled clinical trial to assess daily oral TDF, oral TDF/FTC, and 1% tenofovir vaginal gel for HIV prevention among women in South Africa, Uganda, and Zimbabwe [1] | **Purpose:** To explore adherence challenges (VOICE D)  **Methods:**   - In-depth interviews (IDIs) were conducted with a sub-set of trial participants with varying plasma TFV data levels (i.e., low/inconsistent and high) from all three participating countries. - Focus group discussions (FGDs) were conducted with trial participants with lower adherence. - A pictorial tool was used to show participants their plasma results as a method to facilitate discussion about the challenges with study product adherence.   **Findings:**   - Using participants’ narratives on adherence challenges, a typology of study product use among trial participants was created: - Non-initiation (i.e., never took the study product). - Discontinuation (i.e., temporary or permanent non-use of the study product). - Mis-implementation (i.e., took study product but incorrectly). - Adherence (i.e., took study product as instructed). - Concerns about side effects also influenced adherence [2]. - Based on the evidence gained by showing pharmacokinetic data to trial participants, the VOICE D study team recommended providing more objective adherence feedback to participants as a means to facilitate more accurate discussions about adherence [3].   **Purpose:** To explore the misreporting of study product use (VOICE D).  **Methods:** IDIs and FGDs were conducted with trial participants in two stages of data collection.  **Findings:** Five main reasons were identified for over-reporting of study product adherence:   1. Lying is an intrinsic human trait. 2. Self-preservation motivations. 3. Participants’ concerns about negative consequences for non-adherence. 4. Participants were not caught lying. 5. It was easy to do and less burdensome [4]. |
| CONRAD’s randomized, double-blind, placebo-controlled clinical trial of vaginal gel cellulose sulfate [5] | **Purpose:** To explore study gel acceptability and adherence to the study gel during the trial.  **Methods:** IDIs were conducted with trial participants during exit post-trial interviews.  **Findings:**   - Multiple levels of influence impacted adherence. - Among other findings, participants reported liking the gel, especially because of its lubricant qualities. - Consistent use of the gel proved difficult, particularly due to partner dynamics: gel use was purported to be more common and possible during sex with paying clients compared to sex with primary partners, where perceptions of HIV risk were low and concerns about partner disapproval discouraged use. - Social scientists concluded that gel acceptability alone cannot lead to gel adherence; the role of interpersonal and contextual factors must be considered in future microbicide gel clinical trials [6]. |
| Methods for Improving Reproductive Health in Africa (MIRA) clinical trial—an effectiveness study of the Ortho all-flex diaphragm [7] | **Purpose:** To explore factors influencing adherence to the combined use of the diaphragm/gel and condoms.  **Methods:**   - FGDs were conducted with exiting trial participants - IDIs and FGDs were conducted with sexual partners of trial participants   **Findings:**   - Men controlled the timing of sex, making it challenging for trial participants to use the diaphragm consistently. - Many trial participants viewed the diaphragm as an effective HIV prevention method; hence, the diaphragm was often used as a back-up HIV prevention method in situations where trial participants’ sexual partners refused condom use. - The independent use of the diaphragm and condoms rotated based on which product was available, leading investigators to suggest that multiple prevention methods are needed and desired [8]. |

^1^The BSSR studies may have had other objectives than those listed here.

**References:**

1. Marrazzo JM, Ramjee G, Richardson BA, Gomez K, Mgodi N, Nair G, et al. Tenofovir-based preexposure prophylaxis for HIV infection among African women. N Engl J Med. 2015;372(6):509-18.
2. van der Straten A, Montgomery ET, Musara P, Etima J, Naidoo S, Laborde N, et al. Disclosure of pharmacokinetic drug results to understand nonadherence. AIDS. 2015;29(16):2161-71.
3. Musara P, Montgomery ET, Mgodi NM, Woeber K, Akello CA, Hartmann M, et al. How presentation of drug detection results changed reports of product adherence in South Africa, Uganda and Zimbabwe. AIDS Behav. 2017.
4. Montgomery ET, Mensch B, Musara P, Hartmann M, Woeber K, Etima J, et al. Misreporting of product adherence in the MTN-003/VOICE trial for HIV prevention in Africa: participants' explanations for dishonesty. AIDS Behav. 2017;21(2):481-91.
5. Van Damme L, Govinden R, Mirembe FM, Guedou F, Solomon S, Becker ML, et al. Lack of effectiveness of cellulose sulfate gel for the prevention of vaginal HIV transmission. N Engl J Med. 2008;359(5):463-72.
6. Greene E, Batona G, Hallad J, Johnson S, Neema S, Tolley EE. Acceptability and adherence of a candidate microbicide gel among high-risk women in Africa and India. Cult Health Sex. 2010;12(7):739-54.
7. Padian NS, van der Straten A, Ramjee G, Chipato T, de Bruyn G, Blanchard K, et al. Diaphragm and lubricant gel for prevention of HIV acquisition in southern African women: a randomised controlled trial. Lancet. 2007;370(9583):251-61.
8. Kacanek D, Dennis A, Sahin-Hodoglugil NN, Montgomery ET, Morar N, Mtetwa S, et al. A qualitative study of obstacles to diaphragm and condom use in an HIV prevention trial in sub-Saharan Africa. AIDS Educ Prev. 2012;24(1):54-67.
